# Are oral deformities in tadpoles accurate indicators of anuran chytridiomycosis?

**DOI:** 10.1371/journal.pone.0190955

**Published:** 2018-01-10

**Authors:** Alba Navarro-Lozano, David Sánchez-Domene, Denise C. Rossa-Feres, Jaime Bosch, Ricardo J. Sawaya

**Affiliations:** 1 Departamento de Zoologia e Botânica. Universidade Estadual Paulista, São José do Rio Preto, São Paulo, Brazil; 2 Instituto de Pesquisa em Bioenergia, Universidade Estadual Paulista, Rio Claro, São Paulo, Brazil; 3 Museo Nacional de Ciencias Naturales, CSIC, Madrid, Spain; 4 Centro de Ciências Naturais e Humanas, Universidade Federal do ABC, São Bernardo do Campo, São Paulo, Brazil; Universitat Trier, GERMANY

## Abstract

We evaluated the use of oral deformities as reliable proxies for determining *Batrachochytrium dendrobatidis (Bd)* infection in tadpoles of six anuran species of the Atlantic Forest in southeastern Brazil. We examined oral discs of 2156 tadpoles of six species of anurans collected in 2016: *Aplastodiscus albosignatus*, *Boana albopunctata*, *Boana faber*, *Scinax hayii*, *Crossodactylus caramaschii*, and *Physalaemus cuvieri*. Three oral deformities were recognized: lack of keratinization only in upper and/or lower jaw sheaths, lack of keratinization only in upper or lower tooth rows, and both deformities together. A subsample composed of all the individuals possessing oral deformities (N = 195) plus randomly selected individuals without oral deformities (N = 184) were tested for *Bd* via qPCR. Oral deformities were observed in all six species, but only five were infected with *Bd*. Since we found that dekeratinization of tooth rows was not associated with the presence of *Bd* in any of the studied species we used a new proxy (jaw sheaths dekeratinization with or without dekeratinization in tooth rows: JSD-proxy) for *Bd* detection. Our results showed a nonrandom relationship between *Bd* infection and JSD-proxy in three species of the family Hylidae. However, the use of JSD-proxy for *Bd* detection in these species resulted in up to 30.8% false positives and up to 29.3% false negatives. The use of the JSD-proxy in species for which no relationship was found reached 100% of false positives. We conclude that the use of oral dekeratinization as a generalized proxy for *Bd* detection in tadpoles should not be used as a single diagnosis technique.

## Introduction

Global loss of biodiversity is one of the most serious problems of our time, with Amphibia being the most affected vertebrate class [1, 2]. Habitat loss, introduction of exotic species, environmental contamination, increased UV radiation, climate change and infectious diseases have been identified as major causes of amphibian population declines [3–17]. Chytridiomycosis, an infectious fungal disease caused by *Batrachochytrium dendrobatidis* (*Bd*) [15], has been linked to many incidents of amphibian mass mortality worldwide [15,18,19], and is considered one of the greatest causes of global amphibian declines [20]. In amphibians, *Bd* infection occurs only in keratinized tissues, which are restricted to the oral region (jaw sheaths and teeth) of tadpoles, and the epidermis of metamorphs and adults [15, 21–24].

Several studies have established a relationship between *Bd* infection and the occurrence of anomalies in the oral region of tadpoles of a number of different amphibian species. Fellers et al. [25] found that 67% of *Rana muscosa* tadpoles with abnormally keratinized mouthparts were infected by *Bd*. In turn, Knapp and Morgan [26] found for the same species that 89% of tadpoles with less than 90% jaw sheath pigmentation were infected. This relationship was later reinforced by Drake et al. [27], who found clear nonrandom relationships between oral deformities and *Bd* presence in *Lithobates sphenocephalus* (= *Rana sphenocephala)* tadpoles. A similar result was reported for *Hylodes japi* tadpoles, for which 94.5% of infected individuals possessed depigmented mouthparts [28]. These results suggest the possibility of using tadpole oral deformities as a general proxy for *Bd* detection in tadpoles, as has been previously suggested by Fellers et al. [25] and recently applied by Carvalho et al. [29].

Despite this promising proxy for which only a quick visual inspection is required, there are many studies that have indicated that oral deformities are not always related to *Bd* infection. For example, no relationship was found between depigmentation of mouthparts and *Bd* infection of *Lithobates catesbeianus (= Rana catesbeiana)* and *Pseudacris regilla* [30]. Furthermore, a laboratory study found no differences in the proportions of tadpoles with mouthpart abnormalities between *Bd* infected and uninfected individuals of *Anaxyrus boreas* (= *Bufo boreas*) and *Pseudacris regilla (= Hyla regilla)* [31]. In addition, many other factors have been associated with oral deformities in tadpoles, including low temperatures [32], water contamination [33], nutrition [21], competition [34], and predation risk [35]. Herein we quantitatively evaluate, in detail, the use of oral deformities as a reliable proxy for determination of *Bd* infection in six anuran species from southeastern Brazil.

## Material and methods

### Ethics statement

Field studies did not involve endangered or protected species.

Tadpoles were captured in accordance with collection permits, and subsequently killed using lidocaine, and preserved in 90% alcohol for subsequent study of oral deformities. Collection permits were provided by Instituto Chico Mendes de Conservação da Biodiversidade (ICMBio) (#47148–2). All sampling procedures were reviewed and specifically approved by ICMBio and Comissão Técnico-Científica do Instituto Florestal (COTEC; a committee of Instituto Florestal, the state research agency and responsible for the reserve) (Processo SMA #260108–001.809/2015).

### Study animals

A total of 2156 tadpoles were studied. External morphology and tooth rows formulae were used to identify the species of tadpoles. Tadpoles were collected by dip netting during February (2016) in Núcleo Curucutu (23^o^ 59’ 08.52”S, 46^o^ 44’ 35.76” W), Parque Estadual da Serra do Mar (São Paulo State, Brazil), an understudied old growth area of Atlantic Forest in southeastern Brazil [36].

### Identification of oral deformities

It is important to emphasize that studies regarding the relationship between *Bd* infection and oral deformities have used different, and sometimes ambiguous terms for describing the deformities. For example, while some of studies describe oral deformities as depigmentation of mouthparts (e.g. [28, 30]), others refer to them as dekeratinization (e.g. [27, 31]). This ambiguity was recognized by Altig [37], who pointed out that the “depigmentation” as described in some studies was, in fact, dekeratinization of mouthparts. Therefore, we have chosen to use the term dekeratinization of mouthparts.

**Fig 1:**
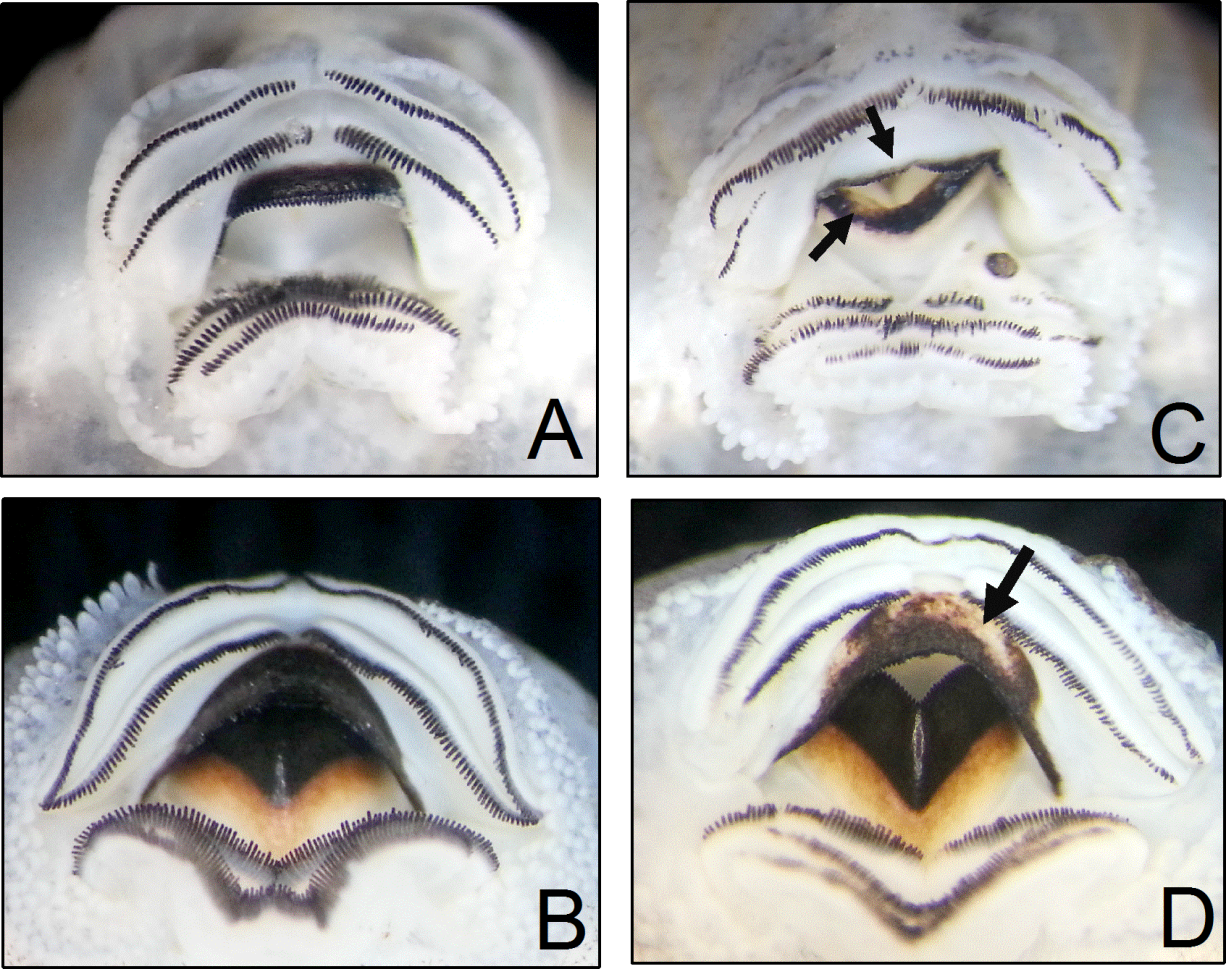
Example of oral deformities found among the studied tadpoles. (A) and (B) = normal tadpoles; (C) = tadpole with dekeratinized jaw sheaths (black arrows) and generalized dekeratinization in tooth rows; (D) = tadpole with dekeratinized upper jaw sheath (black arrow). Upper images *Boana albopunctata*, and lower images *Scinax hayii*.

All individuals studied were between Gosner stages 25 and 40, since the oral disc of tadpoles within these stages normally possesses completely keratinized tissues [24, 38]. Oral discs of tadpoles were examined in detail using a stereoscopic microscope (Leica 54 MZ75), and tadpoles were classified in accordance with dekeratinization (from partial to complete absence of keratinized structures) that presented in mouthparts: **JS** (tadpoles with lack of keratinization only in upper and/or lower jaw sheath), **TR** (tadpoles with lack of keratinization, with non-disrupted supporting tissue, only in upper and/or lower tooth rows), and **JT** (tadpoles with lack of keratinization in jaw sheaths and tooth rows) (Fig 1). All tadpole specimens were deposited in the Amphibia—Tadpoles collection of the Department of Zoology and Botany of UNESP–São José do Rio Preto (DZSJRP-Amphibia-Tadpoles).

### *Batrachochytrium dendrobatidis* detection

After the oral discs of all tadpoles were inspected, a subsample, composed of all the individuals that possessed oral deformities (N = 195) and 184 randomly selected individuals without oral deformities, was analyzed for *Bd* detection. Oral discs of selected tadpoles were excised and air-dried on filter paper as described in Hyatt et al. [39]. DNA oral disc extraction was performed using PrepMan Ultra (Applied Bioysistems) and amplified using a CFX96^TM^ Real-Time PCR Detection System (Bio-Rad) with a *Bd*-specific Taqman Assay [40]. Each 96-well assay plate included a negative control and four different standards containing DNA from 100, 10, 1 and 0.1 *Bd* genome equivalents. For all samples, the negative control and standards were run in duplicate. Samples that showed signs of inhibition (nonsigmoidal amplification) were further diluted to 1:100 and re-analyzed. Only the presence or absence of *Bd* was determined, and samples were considered *Bd* positive when both of the two duplicate analyses revealed *Bd* zoospore genome equivalents >0.1, and the amplification curves have a sigmoidal shape. If not, the sample was re-run and considered positive only with another positive result recorded. DNA analyses were carried out at Museo Nacional de Ciencias Naturales, CSIC (Madrid, Spain).

### Statistical analyses

Two-tailed Fisher’s exact test was used to assess the association between individuals with oral deformities and infection with *Bd* for the total number of tadpoles analyzed for *Bd* presence of all six species sampled. We constructed contingency tables (2 x 2) with two binary variables: 1 = oral deformities present, 0 = oral deformities not present; and 1 = *Bd* infection, and 0 = no *Bd* infection.

## Results

The tadpoles studied belonged to six different species: *Aplastodiscus albosignatus* (N = 721), *Boana albopunctata* (N = 793), *Boana faber* (N = 467), *Scinax hayii* (N = 80), *Crossodactylus caramaschii* (N = 34) and *Physalaemus cuvieri* (N = 61). At least one type of oral deformity was found in 9% (195) of the 2156 tadpoles inspected. Of the tadpoles that exhibited oral deformities, 51.3% had JT, while 29.2% had only TR, and 19.5% only JS.

There was no relationship between *Bd* infection and tooth rows dekeratinization (TR) in any of studied of species (Table 1). However, dekeratinization of the jaw sheath, accompanied or not with dekeratinization of teeth (JS or JT), was related to *Bd* infection in three of the species studied: *A*. *albosignatus* showed a nonrandom relationship with JS (Fisher’s exact test; N = 197, two-tailed p = 0.0023) and JT (N = 197, two-tailed p < 0.001); *B*. *albopunctata* showed nonrandom relationship with JT (N = 63, two-tailed p < 0.001); and *S*. *hayii* showed nonrandom relationships with JS (N = 32, two-tailed p = 0.0068). By contrast, *B*. *faber*, *C*. *caramaschii* and *P*. *cuvieri* did not exhibit relationship between any of the oral deformities studied and *Bd* infection (Table 1).

**Table 1:** Oral deformities and related *Batrachochytrium dendrobatidis (Bd)* infections of tadpoles of six species of Atlantic Forest anurans in southeastern Brazil.

				JS			TR			JT			JSD-Proxy		
	Ns	Na	N_Bd+_	Nd	N_Bd+_	P-value	Nd	N_Bd+_	P-value	Nd	N_Bd+_	P-value	Nd	N_Bd+_	P-value
***Aplastodiscus albosignatus***	721	197	124	27	24	**0.0023**	25	6	< 0.001^a^	78	73	**< 0.001**	105	97	**< 0.001**
***Boana albopunctata***	793	63	12	1	1	0.1904	20	0	0.0124^a^	12	8	**< 0.001**	13	9	**< 0.001**
***Boana faber***	467	33	8	2	2	0.0530	5	1	0.9999	0	0	0.9999	2	2	0.0530
***Scinax hayii***	80	32	16	7	7	**0.0068**	2	1	0.9999	4	4	0.1012	11	11	**< 0.001**
***Crossodactylus caramaschii***	34	34	5	1	0	0.9999	2	0	0.9999	5	2	0.1464	6	2	0.2053
***Physalaemus cuvieri***	61	20	1	0	0	0.9999	3	0	0.9999	1	0	0.9999	1	0	0.9999
**TOTAL**	2156	379	158	38	34	**< 0.001**	57	8	< 0.001^a^	100	87	**< 0.001**	138	121	**< 0.001**

Significant p-values of two tailed Fisher’s exact tests (in bold) indicate a non-random relationship between presence of oral deformities and *Bd* infection. **Ns** = tadpoles studied; **Na** = tadpoles analyzed for *Bd* detection; **N**_**Bd+**_ = tadpoles infected by *Bd*; **Nd** = tadpoles with oral deformities corresponding to **JS** = lack of keratinization only in jaw sheaths, **TR** = lack of keratinization, with non-disrupted supporting tissue, only in tooth rows, **JT** = lack of keratinization in jaw sheaths and tooth rows, and **JSD-Proxy** = jaw sheath dekeratinization with or without dekeratinization in tooth rows.

^a^ Significant relationship but in the opposite direction (absence of TR deformity related to presence of *Bd*).

In the light of these results, we decided to sum JS and JT into a single proxy, JSD-proxy (JSD = jaw sheaths dekeratinization with or without dekeratinization in tooth rows), in order to simplify the determination of oral dekeratinization of tadpoles in future studies. Same statistical analyses performed for TR, JS and JT were then done for this JSD-proxy. The results showed that JSD-proxy considerably improved the accuracy of identifying *Bd* infection. Our original proxies (JS, TR and JT) included 33.8% false positives and 20.1% false negatives for *Bd* infection; the JSD-proxy, on the other hand, included only 12.3% false positives and 18.7% false negatives (see details in Table 2). False positives corresponded to tadpoles that possessed oral deformities but were negative for *Bd* infection via qPCR analysis. False negatives, on the other hand, corresponded to tadpoles that possessed normal oral mouthparts but were positive for *Bd* infection via qPCR.

**Table 2:** Cases of false positives and false negatives for the detection of *Batrachochytrium dendrobatidis (Bd)* infection produced by the use of the three oral deformities studied as proxies (JS, TR and JT) and for the JSD-proxy.

	JS, TR and JT						JSD-Proxy					
	Nd	False positives		Nn	False negatives		Nd	False positives		Nn	False negatives	
		n	%		n	%		n	%		n	%
*Aplastodiscus albosignatus*	130	27	20.8	67	21	31.3	105	8	7.6	92	27	29.3
*Boana albopunctata*	33	24	72.7	30	3	10.0	13	4	30.8	50	3	6.0
*Boana faber*	7	4	57.1	26	5	19.2	2	0	0.0	31	6	19.3
*Scinax hayii*	13	1	7.7	19	4	21.1	11	0	0.0	21	5	23.8
*Crossodactylus caramaschii*	8	6	75.0	26	3	11.5	6	4	66.7	28	3	10.7
*Physalaemus cuvieri*	4	4	100	16	1	6.25	1	1	100	19	1	5.26
**TOTAL**	195	66	**33.8**	184	37	**20.1**	138	17	**12.3**	241	45	**18.7**

**Nd** = tadpoles with oral deformities corresponding to **JS** = lack of keratinization only in jaw sheaths, **TR** = lack of keratinization, with non-disrupted supporting tissue, only in tooth rows, **JT** = lack of keratinization in jaw sheaths and tooth rows, and **JSD-Proxy** = jaw sheath dekeratinization with or without dekeratinization in tooth rows; **Nn** = normal tadpoles.

Although the use of JSD-Proxy was more accurate at identifying *Bd* infection in tadpoles than our original proxies, the analyses of the relationships between *Bd* infection and JSD-proxy found a nonrandom relationship only for three species of the family Hylidae: *A*. *albosignatus* (Fisher’s exact test; N = 197, two-tailed p < 0.001), *B*. *albopunctata* (N = 63, two-tailed p<0.001), and *S*. *hayii* (N = 32, two-tailed p < 0.001) (see details in Table 1).

## Discussion

This is the first study to report the presence of *Bd* in Núcleo Curucutu, one of the largest and most preserved remnants of Atlantic Forest in São Paulo State, which is also a refuge to one of the most biodiverse amphibian faunas of a single locality [41]. Therefore, this virtually pristine area is added to the many others around the world that possess *Bd* (e.g. [18, 42]).

Not all of the tadpoles that possessed oral deformities were infected by *Bd*, and so some of the factors that caused the deformities in our studied tadpoles remain unknown and deserve further investigation. Since our study area is a very well preserved remnant of Atlantic Forest, we considered that one might rule out chemical contamination as the cause of deformities. More importantly, our results showed that TR was the worst proxy for *Bd* infection; in fact, of the 57 tadpoles exhibited only TR deformities, only 8 (14%) were infected with *Bd*. On the other hand, of the 138 tadpoles possessing dekeratinization of the jaw sheaths, accompanied or not with dekeratinization of the teeth, 121 (87.7%) were infected. These results are similar to those obtained by Knapp and Morgan [26] and Vieira et al. [28], who also found a strong correlation between jaw sheaths dekeratinization and *Bd* infection. This finding is also consistent with Marantelli et al. [24], who found that the dekeratinization of jaw sheaths indicates a heavier infection than dekeratinization of tooth rows.

Despite the fact that the analyses of all studied tadpoles together found a strong relationship between tadpoles possessing dekeratinization of jaw sheaths and *Bd* infection, the results by species were less clear. Only three species of the family Hylidae exhibited a significant relationship between JSD-Proxy and *Bd* infection, which was not observed for *B*. *faber* (Hylidae), *C*. *caramaschii* (Hylodidae) and *P*. *cuvieri* (Leptodactylidae). These results agree with Blaustein et al. [31], who also detected varying relationships between *Bd* infection and oral deformities among species. Therefore, and in discordance with Padget-Flohr and Goble [30], who concluded that *Bd* infection and anuran larval mouthpart deformities are two separate processes, we found that a relationship between dekeratinization in jaw sheaths and *Bd* infection does indeed exist in three species of family Hylidae.

Although the relationship between jaw sheaths and *Bd* infection was found for some of our studied species, the use of JSD-proxy remains unreliable for purposes of assessing *Bd* prevalence in tadpoles populations. Based on this proxy, *Bd* detection in species that exhibited a positive relationship with jaw sheaths dekeratinization resulted in up to 30.8% false positives in *B*. *albopunctata* and up to 29.3% false negatives in *A*. *albosignatus*. Furthermore, the use of the JSD-proxy in species for which no relationship was found, reached 100% of false positives. Further illustrating the unreliability of JSD-proxy, in an open area pond where we sampled 23 tadpoles of *B*. *albopunctata* (two of them with dekeratinized jaw sheaths), the use of JSD-proxy would have classified the pond as a *Bd* positive site although qPCR analyses did not confirm this assumption.

Thus, we conclude that the use of oral dekeratinization as a generalized proxy for *Bd* detection in tadpoles should not be used as a single diagnosis technique. We then recommend the use of more accurate techniques such as histology, histochemistry, or PCR analyses in order to obtain accurate diagnosis. However, it is worth noting the usefulness of using JSD-proxy as a screening tool in those species for which the relationship between *Bd*-infection and oral dekeratinization has been proven, since its use minimize the number of individuals evaluated by more costly or time-intensive methods.
